# H-VIP: quantifying regional topological contributions of the brain network to cognition

**DOI:** 10.3389/fradi.2025.1686780

**Published:** 2025-12-04

**Authors:** Sumita Garai, Sandra Vo, Lucy Blank, Frederick Xu, Jiong Chen, Duy Duong-Tran, Yize Zhao, Brielin C. Brown, Li Shen

**Affiliations:** 1 Department of Biostatistics, Epidemiology and Informatics, University of Pennsylvania, Philadelphia, PA, United States; 2 Department of Biology, Regis University, Denver, CO, United States; 3 Department of Biostatistics, Yale University, New Haven, CT, United States

**Keywords:** persistent homology, Homological Vertex Importance Profile, cognition, neuroimaging, brain network

## Abstract

**Introduction:**

Understanding the role of various brain regions of interest (ROIs) in various cognitive functions or tasks, across healthy or neurodegenerative conditions and multiple degrees of separation, remains a key challenge in neuroscience. Conventional network measures can only capture localized or quasi-localized features of brain ROIs. Topological data analysis (TDA), particularly persistent homology, provides a threshold-free, mathematically rigorous framework for identifying topologically salient features in complex networks. In this paper, we introduce a new metric, the Homological Vertex Importance Profile (H-VIP), designed to assess the relevance of vertices that participate in persistent topological structures (e.g., connected components, cycles or cavities) in brain networks. The H-VIP quantifies the topological features of the network at the ROI (node) level by compressing its higher-order connectivity profile using homological constructs.

**Methods:**

Leveraging homological constructs of brain connectomes, we extend two of our previously defined network-level measures—average persistence and persistence entropy—to an ROI-level measure, i.e., the H-VIP. We then applied the H-VIP to two independent datasets: structural connectomes from the Human Connectome Project and functional connectomes from the Alzheimer’s Disease Neuroimaging Initiative. Persistent homology was computed for each network, and H-VIP scores were derived to evaluate vertex-level contributions. Finally, H-VIP scores were used for the prediction of multiple cognitive measures.

**Results:**

In both anatomical and functional brain networks, H-VIP values demonstrate predictive power for various cognitive measures. Notably, the connectivity of the frontal lobe exhibited stronger correlations with cognitive performance than the whole-brain network.

**Discussion:**

H-VIP offers a robust and interpretable means to locate, quantify, and visualize region-specific contributions to network’s topological, higher-order landscape. Its ability to detect potentially impaired connectivity at the individual level suggests possible applications in personalized medicine for neurological diseases and disorders. Beyond brain connectomics, the H-VIP can be used for other types of complex networks where topological features are of importance, such as financial, social, or ecological networks.

## 1 Introduction

Understanding the neurological underpinnings that give rise to higher-order cognitive functions of the brain remains a long-standing goal of neuroscience. By addressing the question of how neural circuits control or affect cognitive activities, one can advance treatment and prevention plans for various diseases and disorders that induce cognitive malfunction, such as Alzheimer’s disease [1], fronto-temporal dementia [2], Parkinson’s disease [3], traumatic brain injury [4], attention deficit disorder [5, 6], and other forms of cognitive impairments. A robust understanding of cognitive processes can also help healthy adolescents and adults optimize memory, learning, productivity, and the smooth execution of their day-to-day cognitive tasks.

Modern tools such as functional neuroimaging, tractography, electrophysiology, and cognitive genomics have proven useful in uncovering the functions and dynamics of the brain network [7] and are studied under the subfield of network neuroscience [8–10]. The human cortex is parcellated into disjoint brain ROIs, which form the nodes or vertices of the network. Typically, in diffusion MRI (dMRI), structural connectivity edges between pairs of ROIs are defined by measures such as fiber length, number of fibers, fiber anisotropy, and fiber density. In contrast, functional connectomes are represented by the correlations of activity between different brain regions, derived from functional magnetic resonance imaging (fMRI). Connectomics analysis is one of the very few non-invasive, *in vivo* methods available for analyzing the human brain. One of the limitations of connectomics analyses is the lack of consensus on parcellation schemes and thresholding parameters, thereby limiting the consensus on result interpretation [11–14]. In the past decade, topological data analysis tools, such as persistent homology, have proven to be a helpful solution for conducting threshold-free network analysis, especially in the context of brain connectomics [15–20]. Persistent homology offers insights into network connectivity through the understanding of connected components, cycles, and cavities (zero-dimensional, one-dimensional, and two-dimensional persistent homology, respectively).

To explore the connection between persistent cycles and cognition, in a previous study [21], we defined two network-level summary statistics: **a**verage persistence and **p**ersistence entropy. We found these measures to have remarkable predictive power for cognitive performance. Using only two features (average persistence and persistence entropy) for each participant, we noted a substantial correlation between the predicted cognitive performance and the actual cognitive score. In this study, we complement our previous work by quantifying local topological contribution from each region, thus providing a new metric which can help with refined understanding of local network topology. We propose a new metric, the Homological Vertex Importance Profile (H-VIP), for the brain network of each participant, which yields a vector whose length is equal to the size of the network, with each component representing the significance score of each ROI in the network. The H-VIP metric proposed here is an attempt to provide a top-down or global-to-local measure for understanding topological cycles in a network. In essence, this metric captures and compresses connectivity information from nodes at *multiple degrees of separation* and projects this information back onto the local nodes.

**Figure 1:**
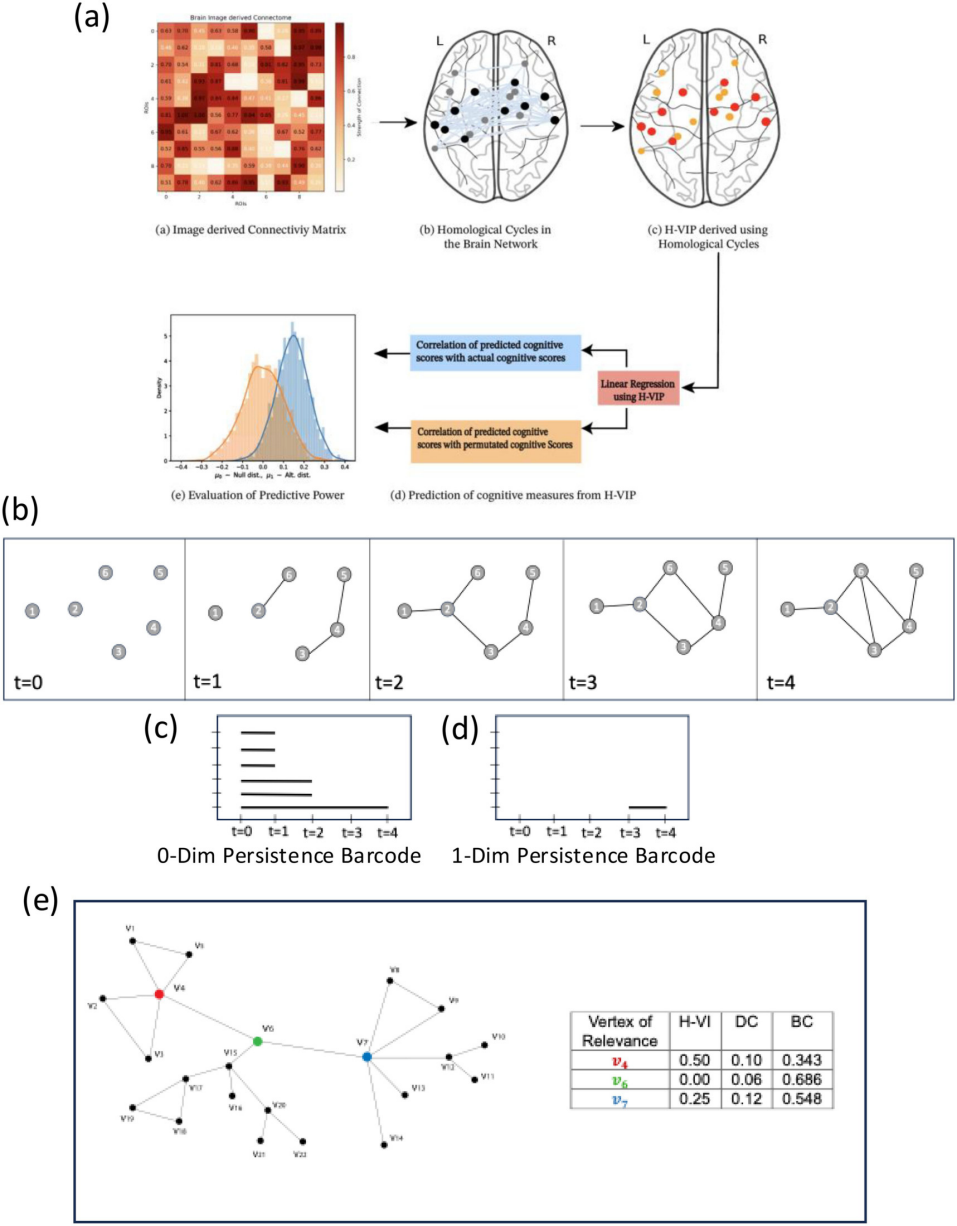
**(a)** Workflow: Prediction of cognitive scores using the Homological Vertex Importance Profile (H-VIP). **(b)** Simplicial filtration of a point cloud showing birth and death of cycle [e.g., **(2,3,4,6)**] at threshold/time t=3 and t=4, respectively. **(c)** Zero-dimensional persistence barcode corresponding to the number of connected components across spatial parameters/filtration. **(d)** One-dimensional persistence barcode corresponding to the number of cycles with their birth/death times across spatial parameters/filtration. **(e)** Example graph illustrating the distinction among various metrics: homological vertex importance (H-VI), degree centrality (DC), and betweenness centrality (BC). We note that nodes $V_{4},V_{6}$, and $V_{7}$ are highly relevant for connectivity in the graph. Node $V_{4}$ is most involved in cycles/loops, whereas node $V_{6}$ displays the highest betweenness and $V_{7}$ shows the highest degree. In other words, both DC and BC are *blind* to brain subcircuitry with close loop communications, which is captured by the H-VI metric.

We applied the proposed H-VIP metric to two datasets: (1) whole-brain structural and frontal sub-networks from young, healthy adults in the Human Connectome Project [22], and (2) functional networks derived from fMRI data from the [23]. Using linear regression, we predicted the cognitive scores of participants from H-VIP values and found statistically significant correlations between the predicted and true cognitive scores (refer to Figure 1a to see the workflow). We then compared the predictive power of cognitive scores by the H-VIP metric with two other popular existing metrics—degree centrality (DC) and betweenness centrality (BC) (which also measure the node/vertex-level participation in a network)—and found that H-VIP often outperforms these measures. These results support the hypothesis that topological features of brain networks are associated with cognitive measures such as fluid intelligence. Moreover, H-VIP demonstrated predictive power for cognitive decline in Alzheimer’s disease, while also revealing changes in the vertex importance profile as the disease advances, showing that this metric holds promise in the context of individualized interventions.

## 2 Methods

### 2.1 Data

We analyzed two independent cohorts. The first comprised young healthy adults from the Human Connectome Project – Young Adult (HCP-YA) dataset, which provides high-quality neuroimaging data to characterize brain network organization during early adulthood, a critical period of brain maturation and development [24]. Preprocessed imaging data were accessed via ConnectomeDB, and structural connectomes were extracted following the pipeline in [25]. ROIs were defined using the Desikan–Killiany (DK) atlas [26], consisting of 68 cortical (34 per hemisphere) and 19 subcortical regions. For each ROI pair, streamlines were identified after gray matter dilation, streamline segmentation to preserve complete pathways, and removal of outliers. We obtained 1,065 structural connectomes from the latest HCP-YA release, from which a subset of 399 non-twin, non-sibling participants was selected based on demographic data.

The second cohort used in the preparation of this article was obtained from the Alzheimer’s Disease Neuroimaging Initiative (ADNI) database (http://adni.loni.usc.edu) [27]. The ADNI was launched in 2003 as a public–private partnership led by Principal Investigator Michael W. Weiner, MD. The primary goal of ADNI has been to test whether serial MRI, PET, other biological markers, and clinical and neuropsychological assessment can be combined to measure the progression of mild cognitive impairment (MCI) and early Alzheimer’s disease (AD). All participants provided written informed consent, and the study protocols were approved by the Institutional Review Board (IRB) of each participating site. Up-to-date information about the ADNI is available at www.adni-info.org.

We examined functional fMRI data from 406 participants in the latest ADNI-3 release, processed in-house using the Connectome Mapper [28]. This cohort included participants with cognitively normal (CN), MCI, and AD, with ROIs defined using the Lausanne atlas [29], comprising 99 regions. Demographic and cognitive scores for both cohorts are summarized in Tables 1–3. All procedures were approved by the institutional review boards of participating sites, and data were downloaded and analyzed under approval of the University of Pennsylvania Institutional Review Board.

**Table 1:** Demographics and cognitive measures of the 399 participants (214 males/185 females) for HCP-YA.

Attributes	Range	Mean ± Std. dev.
Age (in years)	22–36	28.51 ± 3.69
Penn matrix reasoning test (PMAT24)	06.00–24.00	17.24 ±4.69
Oral comprehension	86.20–150.70	116.77 ± 10.30
Oral comprehension, age-adjusted	65.02–138.08	106.87 ± 14.46
Picture vocabulary test	92.84–153.08	116.85 ± 9.19
Picture vocabulary test, age-adjusted	69.45–153.08	109.38 ± 14.56
Computerized Penn word memory test	26.00–40.00	35.88 ± 2.79
Pattern composite processing speed	60.09–154.69	115.06 ± 15.96

**Table 2:** Participant demographics: ADNI cohort.

Attributes	CN	MCI	AD
Number	193	158	55
Gender (M/F)	93/100	85/73	30/25
Age (mean ± std)	70.62 ± 6.56	71.77 ± 7.09	74.23 ± 7.24
Education (mean ± std)	16.63 ± 2.38	16.19 ± 2.72	15.56 ± 2.67

**Table 3:** Cognitive measures and brain atrophy measures for the ADNI Cohort.

Attributes	Range	Mean ± Std. dev.
ADAS	0–68	13.99 ± 9.99
CDRSB	0–15	1.50 ± 2.47
RAVLT	0–11	4.68 ± 2.71
MMSE	9–30	27.61 ± 3.50
FDG	1.007–1.433	1.254 ± 0.084
Whole brain	734,116–1,427,440	1,024,937 ± 103,722
Hippocampus	3,549–10,482	6,994 ± 1,129
Entorhinal	982–6,500	3,907 ± 895
Ventricles	8,659–129,863	43,241 ± 22,627
MidTemp	12,172–31,794	20,180 ± 2,812
Fusiform	10,202–27,087	17,885 ± 2,561

### 2.2 Background on persistent homology

Persistent homology [30–32] organizes multiscale topology into an algebraic time-series (a persistence module). This module has a canonical interval decomposition (called the barcode), which offers a framework for quantifying the evolution of topological features (such as connected components, tunnels, and voids) across multiple spatial or scale parameters. The starting point is a *filtration* of finite simplicial complexes

$$\emptyset =K_{a_{0}}\subseteq K_{a_{1}}\subseteq \cdots \subseteq K_{a_{m}}=K$$

indexed by an ordered set $a_{0}<a_{1}<\cdots <a_{m}$ (often real parameters). Typical filtrations arise either from sublevel sets of a function f:X→R of from Vietoris–Rips or Čech complexes built upon point clouds. We fixed a field k and used $H_{p}(\;\cdot \;)=H_{p}(\;\cdot \;;K)$ to represent the simplicial homology in degree p. Functoriality of homology gives linear maps for a≤b:

$$i_{a\to b}\;:\;K_{a}↪K_{b}\;\Rightarrow \;\phi_{a\to b}^{p}\;:\;=H_{p}(i_{a\to b})\;:\;H_{p}(K_{a})⟶H_{p}(K_{b})$$

The family $\left\{H_{p}(K_{a}),\phi_{a\to b}^{p}\right\}$ is the degree p *persistence module*. Formally, if we regard the index set (I,≤) as a poset category, then a persistence module is a functor

$$V\;:\;(I,\leq )⟶{\mathrm{Vect}}_{k},\;a\mapsto V(a),\;a\leq b\mapsto v_{a\to b}$$

with $v_{a\to a}=\mathrm{id}$ and $v_{b\to c}\circ v_{a\to b}=v_{a\to c}$. In our case, $V(a)=H_{p}(K_{a})$ and $v_{a\to b}=\phi_{a\to b}^{p}$.

A p-dimensional homology class $\alpha \in H_{p}(K_{a})$ is considered *born* at parameter a if it does not lie in the image of $H_{p}(K_{a^{-}})$ (the previous stage). It is said to *die* at the smallest parameter b>a for which $\phi_{a\to b}^{p}(\alpha )$ maps to zero. The *interval decomposition* encodes each class by its lifespan [a,b) (or [a,∞) if it never dies inside the filtration). Under mild finiteness/tameness assumptions [e.g., finite filtrations or q-tame modules, i.e., for $s<t,\;\dim_{k}\;(\text{im}(v_{s\to t}))<\infty$], persistence modules over a totally ordered index set admit a canonical decomposition as a direct sum of *interval modules*. Formally,

$$V≅\underset{\;j}{⨁}K_{\left[b_{j},d_{j}\right)}$$

where $k_{\left[b,d\right)}$ is the functor that equals k on indices t∈[b,d) and 0 otherwise, with identity map within the interval and zero map outside of it. The multiset $\{[b_{j},d_{j}){\}}_{j}$ is the *barcode* in degree p; equivalently, one may represent it as a *persistence diagram*, a multiset of points $\left(b_{j},d_{j}\right)$ above the diagonal in $R^{2}$. Computationally, for a finite filtration, one assembles the boundary matrices $\partial_{p}$ ordered by filtration time and performs a matrix reduction (column operations over K) that pairs the formation of p cycles with the annihilation via (p+1) boundaries. Each reduced pivot encodes an interval [b,d); columns without pivots correspond to intervals of the form [b,∞).

Stability is a key feature of persistent homology, which encodes significance. For filtrations arising from sublevel sets of tame functions f,g:X→R, the stability theorem [33] states that the bottleneck distance between the corresponding diagrams is bounded by the sup-norm of the perturbation:

$$d_{B}\left({\mathrm{Dgm}}_{p}(f),\;{\mathrm{Dgm}}_{p}(g)\right)\;\leq \;\|f-g{\|}_{\infty }$$

Thus, barcodes are robust summaries of shape across scales. In applications, long intervals typically signal topological features that persist across many scales (and are hence considered signal), while very short intervals are often treated as noise [19, 30]. The choice of the field k (commonly the finite field $Z_{2}$ or more generally $F_{p}$ is used) affects torsion and computational convenience but not impact the existence of the interval decomposition for k-vector space-valued modules. A detailed discussion on the mathematical foundation of homology and cohomology is given in [34].

### 2.3 Homological Vertex Importance Profile

In this section, we introduce a new metric, called the homological vertex importance (H-VI), to underscore the contribution of each node of the network from a topological perspective. Representative cocycles are elements of the cochain complex corresponding to cohomology classes, serving as the *dual* of homological cycles. A cycle in homology is a chain of simplices whose boundary vanishes (e.g., a closed loop or surface), whereas a cocycle in cohomology is a linear functional on chains whose coboundary vanishes. In persistent homology, representative cocycles identify which simplices support each persistent feature, providing a method to localize and interpret topological structures such as loops or voids. In practice, representative cocycles are computed algorithmically through the matrix reduction of the **c**oboundary operator:

$$\delta^{1}\;:\;C^{1}(K,F_{p})\to C^{2}(K,F_{p})$$

defined by

$$\left(\delta^{1}\phi )([v_{0},v_{1},v_{2}])=\phi ([v_{1},v_{2}])-\phi ([v_{0},v_{2}])+\phi ([v_{0},v_{1}]\right)$$

A list of triples $\{(u_{i},v_{i},c_{i}){\}}_{i=1}^{k}$ represents a cochain $\phi =\sum_{i=1}^{k}c_{i}[u_{i},v_{i}]$. If the cochain satisfies $\delta^{1}\phi =0$, then it is a 1-cocycle; i.e., its coboundary vanishes.

We used the representative cocyles to define homological vertex importance (H-VI) for any given vertex by quantifying its contribution to the 1D persistence occurring in a graph/network. Let $R=\{\phi_{1},\phi_{2},\ldots \phi_{r}\}$ is the set of r unique representative 1D cocycles in K. The homological vertex importance (H-VI) of the vertex v will be the average number of times it appears in all the representative cocyles, i.e.,

$${\mathrm{VI}}_{C}(v)=\mathrm{card}\{\phi \;\;|\;\;v\in \phi \}/r$$ (1)

Note that the metric vertex importance ${\mathrm{VI}}_{C}(v)$ depends on the chosen cycle C and the vertex v. The Homological Vertex Importance Profile or H-VIP of a network is a vector consisting of relative importance of each vertex of the network in forming 1D cocycles. Formally, H-VIP can be defined as follows:

Definition 1. The Homological Vertex Importance Profile of the network G is given by the tuple:

$$H-VIP(G)=\left(\sum_{C}{\mathrm{VI}}_{C}(v_{1}),\sum_{C}{\mathrm{VI}}_{C}(v_{2}),\ldots ,\sum_{C}{\mathrm{VI}}_{C}(v_{n})\right)$$ (2)

where indices in the sum run over all the cocycles C of the network G.

This profile gives an estimate for the significance of the contribution (in forming cycles) of all the nodes/vertices of the graph. Note that this is a vector of dimension n (the number of vertices in the graph). We utilize the Python package Ripser [35] to compute the representative cocycles and extract the individual homological vertex importance using Equation 1 and followed by homological vertex importance profile vector using Equation 2 for the brain network (whole brain and frontal sub-network), and subsequently use these H-VIP vectors for predicting the cognitive scores of the participants, by applying a linear regression model.

## 3 Results

**Figure 2:**
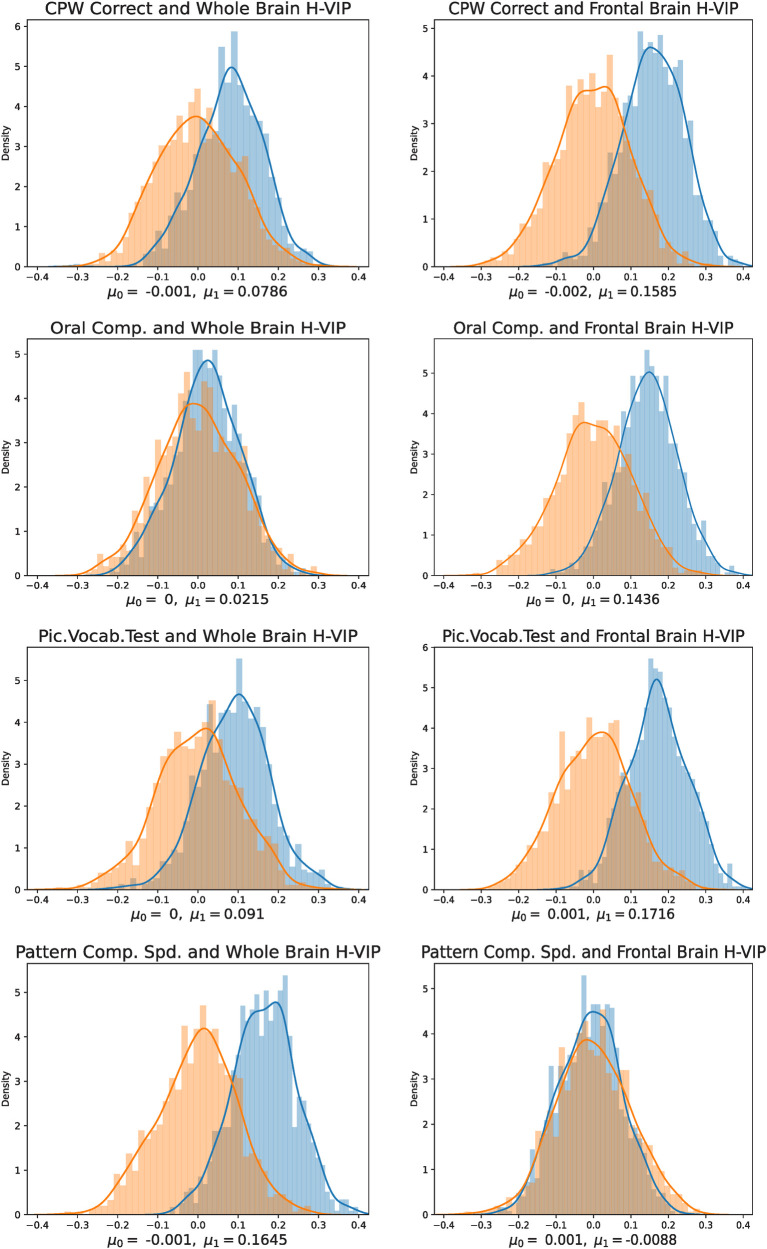
Correlation between H-VIP-predicted cognitive scores and true cognitive scores (show in blue), plotted against the correlation between H-VIP-predicted scores and randomly assigned scores (shown in orange), in the HCP-YA cohort. Here, $\mu_{1}$ represents the mean correlation between predicted and actual scores, and $\mu_{0}$ denotes the mean correlation between permuted scores, each averaged over 1,000 random splits.

**Figure 3:**
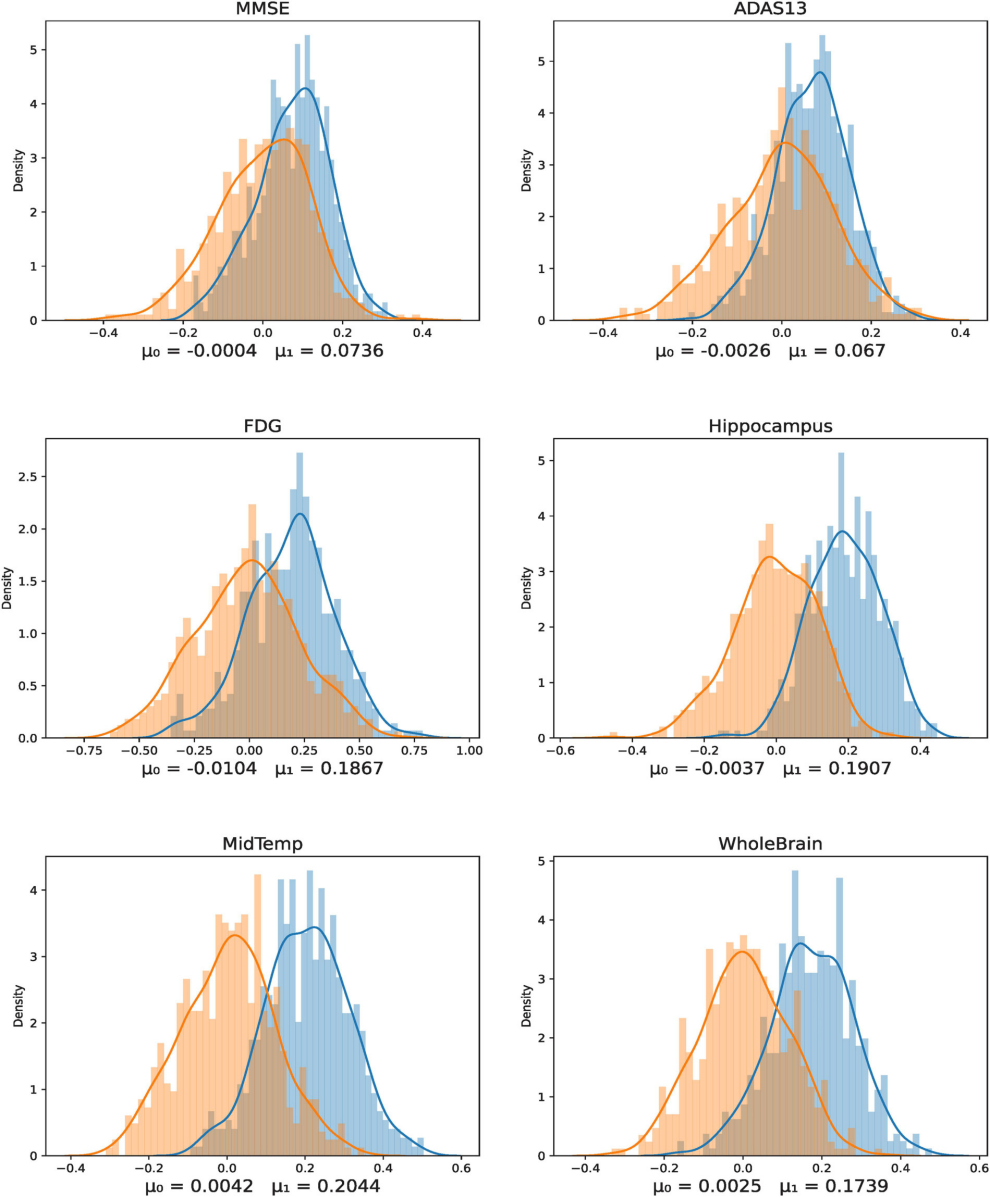
Correlation between predicted and true cognitive scores, biomarkers, and atrophy (shown in blue), plotted against the null distribution of randomly permuted scores (shown in orange), in the ADNI cohort. Here, $\mu_{1}$ represents the mean correlation between predicted and actual scores, and $\mu_{0}$ denotes the mean correlation between permuted scores, each averaged over 1,000 random splits.

For the HCP cohort, we performed multivariate linear regression using the following set of features: (A) Homological Vertex Importance Profile for the whole brain and (B) Homological Vertex Importance Profile for the frontal brain subnetwork, for the cognitive measures listed in Table 1. For the ADNI cohort, we performed the same analysis only for the whole-brain network (as the outcome variable, along with cognitive measures, also includes brain volume and atrophy in various brain regions), see Table 3. For each analysis, we split our participants into two categories: training and testing, in a ratio 80% and 20%, respectively, chosen at random. Subsequently, we performed z-score standardization across participants and measures. For each cognitive measure (or covariate of interest), we used our feature vector H-VIP to train our linear regression model with the training participants (80%). Then, we used the remaining 20% of participants (test participants) to predict the cognitive score and calculate the Pearson correlation between the predicted score and the actual score. The performance is reported on the testing set only, ensuring no overfitting. We repeated this process of splitting data into training and testing participants 1,000 times, each time with a different randomization seed. Each iteration yielded a correlation value, resulting in a normal distribution with a mean of $\mu_{1}$, as shown in blue in Figures 2, 3. Next, we permuted/shuffled (using a randomization seed) the cognitive scores of the 20% test participants and repeated the above process; i.e., we calculated the Pearson correlation between the randomly assigned score and the predicted score, which again yielded another normal distribution, with a mean of $\mu_{0}$, as shown in orange in Figures 2, 3.

Under the null hypothesis, we expect no statistically significant difference between these two distributions. We observe that the correlation between the H-VIP-predicted cognitive scores and permuted (and randomly assigned) cognitive scores forms a normal distribution, with a mean of approximately zero, indicating that the randomly assigned scores are not correlated with the predicted scores. However, we observe a right shift or positive correlation between the predicted scores and true cognitive scores. We also compared graph-theoretic brain connectivity measures, degree centrality (DC) and BC [36], to our proposed measure, H-VIP, to demonstrate its potential. We note that H-VIP is a better predictor of cognitive performance for most of the cognitive measures considered in this study; see Tables 4 and 5, where statistically significant differences are highlighted in bold. For the ADNI study, the outcomes include a set of cognitive assessment tests and physiological biomarkers, such as beta-amyloid, tau, phospho-tau, and atrophy in various regions of the brain, including the whole brain. We observe that whole-brain H-VIP has prediction power for the scores obtained from Alzheimer's Disease Assessment Scale (ADAS), and Mini-Mental State Examination (MMSE), where the highest predictive power is for atrophy in the hippocampus and medial temporal lobe, where we observe the maximum right shift from the null distribution, see Figure 3 and Table 6.

**Table 4:** Correlation between predicted cognitive scores, using the **whole-brain network**-derived Homological Vertex Importance Profile (H-VIP), degree centrality (DC), and betweenness centrality (BC) measures, and the true cognitive scores in the HCP-YA cohort.

Cognitive measure	$\mu_{0}$	$\mu_{1}$	*p*-Value
(a) H-VIP			
PMAT24 correct	0.0026	−0.0033	$9.28\times 10^{-1}$
Oral comp.	0.0005	**0.0215**	$1.53\times 10^{-7}$
Oral comp., age-adj.	0.0008	**0.0192**	$3.63\times 10^{-6}$
Pic.vocab.test	−0.0003	**0.0910**	$2.25\times 10^{-94}$
Pic.vocab.test, age-adj.	−0.0011	**0.0888**	$5.26\times 10^{-94}$
CPW correct	−0.0013	**0.0786**	$7.02\times 10^{-80}$
Pattern comp. spd.	−0.0012	**0.1645**	$2.68\times 10^{-271}$
(b) Degree centrality			
PMAT24 correct	−0.0075	−0.0468	1.00
Oral comp.	−0.0028	**0.0124**	$1.09\times 10^{-4}$
Oral comp., age-adj.	−0.0026	**0.0216**	$1.89\times 10^{-9}$
Pic.vocab.test	0.0034	**0.1394**	6.$01\times 10^{-200}$
Pic.vocab.test, age-adj.	0.0027	**0.1246**	$3.60\times 10^{-168}$
CPW correct	0.0007	0.0025	$3.20\times 10^{-1}$
Pattern comp. spd.	0.0010	**0.1029**	$1.91\times 10^{-123}$
(c) Betweenness centrality			
PMAT24 correct	−0.0012	**0.0818**	$2.38\times 10^{-83}$
Oral comp.	−0.0049	0.0000	$1.31\times 10^{-01}$
Oral comp., age-adj.	−0.0061	**0.0204**	$3.98\times 10^{-10}$
Pic.vocab.test	−0.0006	−0.0059	$9.01\times 10^{-01}$
Pic.vocab.test, age-adj.	−0.0020	**0.0422**	$1.27\times 10^{-25}$
CPW correct	−0.0014	−0.0131	$9.98\times 10^{-1}$
Pattern comp. spd.	0.0002	−0.0912	1.00

Here, $\mu_{1}$ denotes the mean correlation between predicted and actual scores, and $\mu_{0}$ represents the mean correlation between permuted scores, each averaged over 1,000 random splits. Statistically significant differences (p≤0.05, with FDR correction) between $\mu_{0}$ and $\mu_{1}$ are highlighted in bold.

**Table 5:** Correlation between predicted cognitive scores, using the **frontal brain network**-derived Homological Vertex Importance Profile (H-VIP), degree centrality (DC), and betweenness centrality (BC) measures, and the true cognitive scores in the HCP-YA cohort.

Cognitive measure	$\mu_{0}$	$\mu_{1}$	*p*-Value
(a) H-VIP			
PMAT24 correct	0.0006	**0.0498**	$1.84\times 10^{-32}$
Oral comp.	0.0004	**0.1436**	$7.04\times 10^{-213}$
Oral comp., age-adj.	0.0005	**0.1371**	$5.32\times 10^{-196}$
Pic.vocab.test	0.0012	**0.1716**	$7.75\times 10^{-279}$
Pic.vocab.test, age-adj.	0.0017	**0.1770**	$4.33\times 10^{-291}$
CPW correct	−0.0019	**0.1585**	$1.01\times 10^{-249}$
Pattern comp. spd.	0.0006	−0.0088	$9.89\times 10^{-1}$
(b) Degree centrality			
PMAT24 correct	0.0044	−0.0053	$9.91\times 10^{-1}$
Oral comp.	0.0010	**0.0253**	$1.27\times 10^{-9}$
Oral comp., age-adj.	0.0017	**0.0463**	$1.51\times 10^{-27}$
Pic.vocab.test	0.0022	**0.1137**	$4.34\times 10^{-140}$
Pic.vocab.test, age-adj.	0.0018	**0.1440**	$6.62\times 10^{-206}$
CPW correct	−0.0031	−0.0399	1.00
Pattern comp. spd.	−0.0035	**0.0809**	$1.72\times 10^{-86}$
(c) Betweenness centrality			
PMAT24 correct	−0.0053	**0.0523**	$1.96\times 10^{-46}$
Oral comp.	−0.0015	−0.0261	1.00
Oral comp., age-adj.	−0.0034	**0.0197**	$6.10\times 10^{-9}$
Pic.vocab.test	−0.0017	**0.0469**	$4.17\times 10^{-34}$
Pic.vocab.test, age-adj.	−0.0030	**0.0716**	$2.81\times 10^{-71}$
CPW correct	−0.0046	−0.0263	1.00
Pattern comp. spd.	−0.0001	−0.0043	$8.51\times 10^{-1}$

Here, $\mu_{1}$ represents the mean correlation between predicted and actual scores, and $\mu_{0}$ denotes the mean correlation between permuted scores, each averaged over 1,000 random splits. Statistically significant differences (p≤0.05, with FDR correction) between $\mu_{0}$ and $\mu_{1}$ are highlighted in bold.

**Table 6:** Correlation between predicted cognitive scores using the whole-brain network-derived Homological Vertex Importance Profile (H-VIP) and true cognitive scores in the ADNI cohort.

Measure	$\mu_{0}$	$\mu_{1}$	*p*-Value
ADAS13	0.0067	**0.0670**	$7.00\times 10^{-21}$
CDRSB	0.0041	0.0089	$4.72\times 10^{-1}$
RAVLT	0.0020	**0.0504**	$2.50\times 10^{-12}$
MMSE	0.0039	**0.0736**	$5.70\times 10^{-27}$
FDG	0.0010	**0.1867**	$1.82\times 10^{-36}$
WholeBrain	0.0011	**0.1739**	$4.10\times 10^{-106}$
Hippocampus	0.0074	**0.1907**	$1.05\times 10^{-121}$
Entorhinal	0.0035	**0.1059**	$1.27\times 10^{-48}$
Ventricles	0.0002	0.0152	$7.32\times 10^{-2}$
MidTemp	−0.0023	**0.2044**	$1.86\times 10^{-29}$
Fusiform	−0.0050	**0.0858**	$4.61\times 10^{-36}$

Here, $\mu_{1}$ represents the mean correlation between predicted and actual scores, and $\mu_{0}$ denotes the mean correlation with permuted scores, each averaged over 1,000 random splits. Statistically significant differences (p≤0.05, with FDR correction) between $\mu_{0}$ and $\mu_{1}$ are highlighted in bold.

## 4 Discussion

The goal of this study is twofold: to understand and quantify the role (or importance) of each brain region for cognitive tasks in healthy adults and to pinpoint individual anatomical differences in the manifestation of disease pathology, specifically in conditions such as Alzheimer’s disease. Connectivity measurement tools have long been used to investigate network efficiency and clustering. These tools quantify integration and segregation properties in brain networks at multiple scales [9, 37, 38]. Since efficient connectivity of a network ensures smooth transmission of information, it has been hypothesized that it is also related to various forms of intelligence, including fluid, crystallized, and general intelligence [37, 39–41].

**Figure 4:**
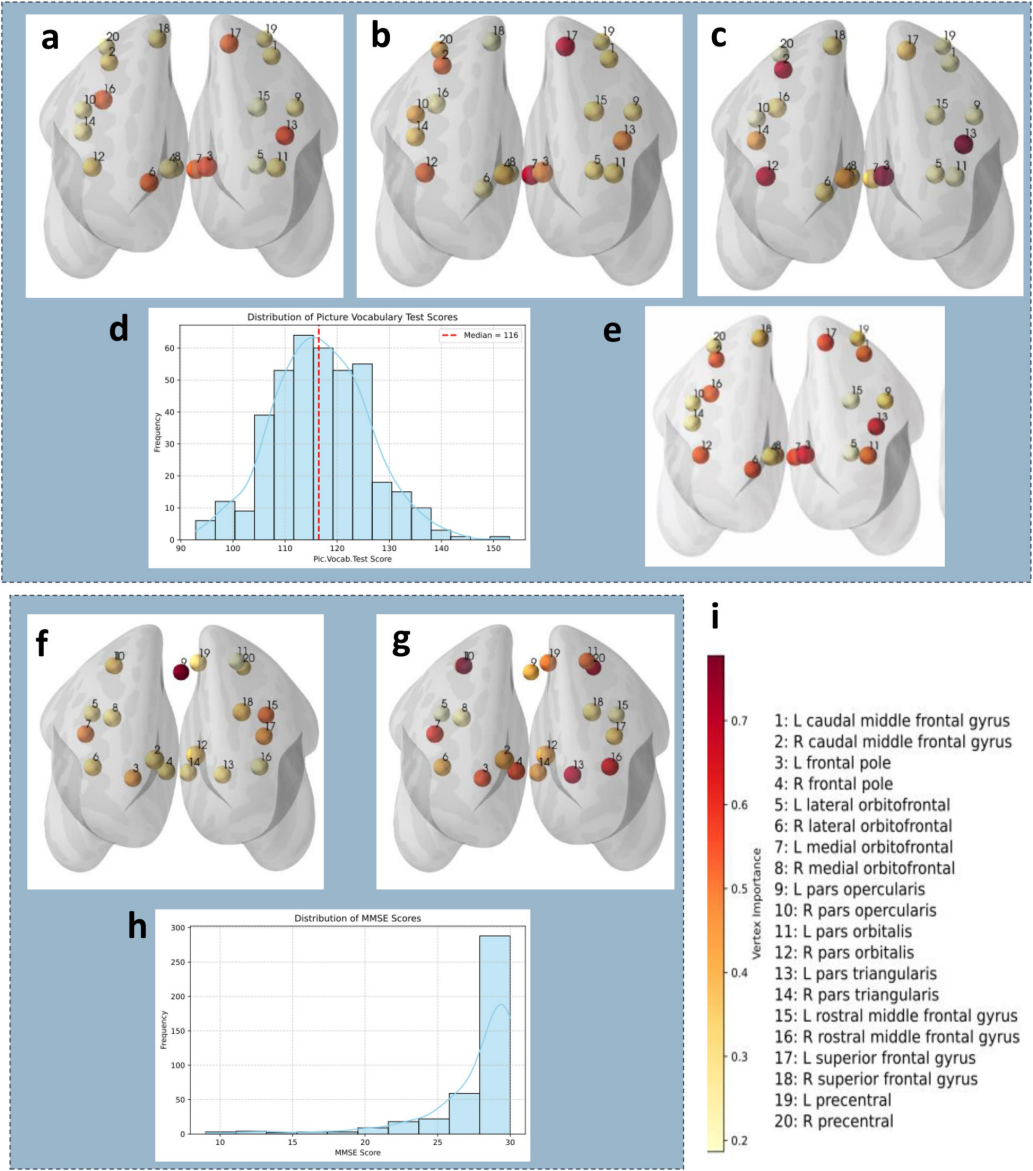
**Top panel: HCP-YA cohort**: H-VIP maps of the frontal brain for participants with **(a)** minimum, **(b)** median, and **(c)** maximum picture vocabulary test scores; **(d)** distribution of picture vocabulary test scores for reference; and **(e)** average H-VIP for 399 participants. **Bottom panel: ADNI-3 cohort**: H-VIP maps of the frontal brain for participants with **(f)** minimum and **(g)** maximum MMSE scores and **(h)** distribution of MMSE scores for reference. The color bar in **(i)** indicates the significance of each ROI (vertex) in the H-VIP framework, and the numbers represent the frontal brain ROIs and labels.

Here, we delve deeper into the underlying mechanism behind information sharing among ROIs, those that are at multiple degrees of separation, through the lens of persistence homology [30, 32]. We device a metric H-VIP to quantify the role of various regions of interest. We find that, on average, the regions in the left frontal pole, left superior frontal gyrus, left pars triangularis, and left medial orbitofrontal cortex have higher vertex importance (Figure 4e). In Figures 4a–c, we compare the frontal subnetwork H-VIP of participants from the HCP-YA cohort with minimum, median, and maximum picture vocabulary scores and note that participants with higher picture vocabulary scores demonstrate higher participation in forming homological cycles, i.e., higher values in the H-VIP, as denoted by the color bar in Figure 4i. In the ADNI cohort, we find that the participant with the lowest MMSE score (Figure 4f) has a distinctive difference in their H-VIP profile compared to the participant with the highest MMSE score (cognitively normal), as shown in Figure 4g. The lower values in the H-VIP profile signify lower participation in homological cycle formation, suggesting impaired brain connectivity.

The frontal pole and superior frontal gyrus are two crucial brain regions located in the frontal lobe of the cerebral cortex. These regions play a critical role in various aspects of cognition, which involve higher-order thinking, decision-making, and goal-directed behavior [42–45]. The medial orbitofrontal cortex (located in the frontal lobes) region has been associated with various cognitive and emotional processes, including decision-making, social behavior, emotion regulation, and reward processing [46], and takes part in the integration of sensory information, emotional responses, and cognitive functions to guide behavior and decision-making. The pars triangularis is one of the three subdivisions of the broader region known as Broca’s area, which is traditionally associated with language production and comprehension [47] but also implicated in working memory and cognitive control, such as regulating attention, inhibiting irrelevant information, and switching between tasks [5, 48]. We also observed that H-VIP and degree centrality of the frontal lobe subnetwork, comprising 20 ROIs, have significantly higher correlations with cognitive performance compared to the whole-brain network. This provides evidence that a distinctive topological structure underlies the frontal brain network correlated with cognitive performance, corroborating the existing literature [49–51].

## 5 Limitations

There are certain limitations inherent to any study of this kind. Estimation of the structural connectivity of the entire brain is notably influenced by data processing parameters, such as the number of streamlines used, different parcellation schemes, various data processing pipelines, and varying resolutions. With due caution, we can still extrapolate behavioral metrics from the anatomical connectivity networks, keeping in mind that our proposed topological characteristics are sensitive to the aforementioned parameters. The functional connectivity network is slightly more complex to interpret because it is built from the coactivation of regions measured from blood-oxygen-level-dependent signals, and it does not have a clear underlying anatomical framework. Despite this constraint, functional networks can effectively monitor areas of brain activity and distinguish areas specific to certain categories of tasks. A relevant future application of H-VIP will be to analyze various task-based (functional) networks and isolate regions of interest activated during specific tasks.

## 6 Conclusions

In this work, we introduce a new metric, called the H-VIP, which provides a formulation of the abstract yet intuitive notion of the degree of participation of individual vertices in forming homological cycles within a graph network. This formulation of H-VIP provides a global-to-local projection of homological relevance of the vertices and can be thought of as an encapsulation of the quasi-local connectivity profile of the network. We applied this metric as a feature vector to predict the cognitive scores of participants from two datasets: structural connectomes from the HCP-YA and functional connectomes from ADNI-3, demonstrating its prediction power. Our study suggests that a higher degree of participation in forming cycles within the structural and functional connectivity networks of the brain is correlated with better cognitive abilities. We conclude with the remark that H-VIP is a metric that can be applied to any general network to infer interesting properties of connectivity and is not limited to brain connectomes.

## Data Availability

Publicly available datasets were analyzed in this study. These data can be found here: https://adni.loni.usc.edu/ https://www.humanconnectome.org/study/hcp-young-adult/data-releases.
